# Ecological responses to experimental glacier-runoff reduction in alpine rivers

**DOI:** 10.1038/ncomms12025

**Published:** 2016-06-24

**Authors:** Sophie Cauvy-Fraunié, Patricio Andino, Rodrigo Espinosa, Roger Calvez, Dean Jacobsen, Olivier Dangles

**Affiliations:** 1 Institut de Recherche pour le Développement (IRD), UMR EGCE, IRD-247 CNRS-UP Sud-9191, Avenue de la Terrasse, Bâtiment 13, 91198 Gif-sur Yvette, France; 2 Pontificia Universidad Católica del Ecuador, Escuela de Ciencias Biológicas, Avenida 12 de Octubre, Quito 170150, Ecuador; 3 IRSTEA, UR MALY, 5 rue de la Doua, Villeurbanne 69100, France; 4 Institut de Recherche pour le Développement (IRD), UMR G-EAU, 361 Rue Jean-François Breton, BP 5095, 34196 Montpellier, France; 5 Freshwater Biological Laboratory, Department of Biology, University of Copenhagen, Universitetsparken 4, 2100 Copenhagen, Denmark

## Abstract

Glacier retreat is a worldwide phenomenon with important consequences for the hydrological cycle and downstream ecosystem structure and functioning. To determine the effects of glacier retreat on aquatic communities, we conducted a 4-year flow manipulation in a tropical glacier-fed stream. Compared with an adjacent reference stream, meltwater flow reduction induces significant changes in benthic fauna community composition in less than 2 weeks. Also, both algal and herbivore biomass significantly increase in the manipulated stream as a response to flow reduction. After the flow reduction ceased, the system requires 14–16 months to return to its pre-perturbation state. These results are supported by a multi-stream survey of sites varying in glacial influence, showing an abrupt increase in algal and herbivore biomass below 11% glacier cover in the catchment. This study shows that flow reduction strongly affects glacier-fed stream biota, prefiguring profound ecological effects of ongoing glacier retreat on aquatic systems.

## Abstract (web summary)

Glacier retreat due to climate change can affect the biodiversity and ecosystem function. Here, Cauvy-Fraunié and colleagues manipulated the flow of tropical glacier-fed stream in the Ecuadorian Andes, and show that benthic fauna community composition requires several months before it can recover to the pre-blockade conditions.

## Graphical abstract

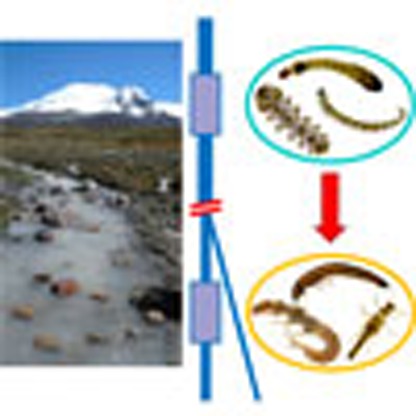

Worldwide recession of glaciers foreshadows global-scale shifts of alpine river flow regimes^1^,^2^. While most glaciers have been shrinking since the end of the Little Ice Age, around 160 years ago, shrinking rates have accelerated over the last 3–4 decades^3^, leading to an increased threat to water resources^1^, alpine biodiversity and associated ecosystem services^4^,^5^,^6^. Indeed, glacial contributions to river flow create a diversity of habitats that are important for a large number of plants and animals^7^, and they support economically important wetlands and fisheries^6^. Moreover, climate-driven fluctuations in discharge following glacier melting are expected to profoundly affect ecological processes in alpine rivers, which are strongly influenced by hydrological and thermal regimes^8^,^9^.

A major issue in elucidating the ecological effects of shrinking glaciers relates to predicting biological community responses to modifications in glacier runoff (for example, lower volumes of ice melt water) and variability (for example, more prolonged and frequent low flows^10^). After a critical threshold of reduction in ice volume, annual glacial runoff should decrease, inducing an increase in low-flow period frequency and intensity followed by continuous low flows until the complete loss of glacier outflow when the glacier disappears^2^. Pronounced decline in glacial discharge observed worldwide is one of the most concerning symptoms of glacier shrinking with unclear consequence on biodiversity^4^. While several studies have shown that many species may become endangered or extinct if glacial meltwater inputs are reduced or entirely lost^4^,^6^,^7^, we still know little about mechanisms driving the response of aquatic biota to glacier retreat so that we can better evaluate their response to global warming^5^,^6^. A whole-ecosystem manipulation of meltwater reduction, simulating the effect of glacier retreat on glacier-fed streams, in which both disturbance effects and return times to pre-disturbance conditions are determined, should provide insights into the ecological response to forthcoming glacial meltwater alteration. Yet such an ecosystem manipulation has never been performed in alpine rivers.

Here we experimentally simulate temporary flow reduction in a glacier-fed stream of the Ecuadorian Andes, a region where a decrease in glacier runoff is expected to be one of the most pronounced worldwide^2^. Using a Before-After-Control-Impacted design^11^, we divert one-third of the glacial meltwater of a glacier-fed stream (manipulated) while an adjacent similar stream (reference) remains undisturbed (see Supplementary Fig. 1 for the field manipulation design). The stream manipulation consists of three time intervals: (1) establishment of baseline conditions under unaltered stream flow (∼1 year); (2) diversion of water flow, inducing a water flow reduction in the downstream reach (∼1 year; the upper reach was kept undisturbed); and (3) reset to initial flow conditions to evaluate post-drought recovery trajectories (∼2 years). This experimental design allows us to assess the response of benthic algal and faunal communities to flow reduction, and evaluate the ecosystem resilience, defined as the capacity of the benthic communities to return to their initial configuration after flow disturbance cease^12^. To determine whether our experimental results would be corroborated across a spatial gradient in glacial cover, we analyse the spatial variability in benthic algae and fauna across 33 stream sites, in the same study area. Corroboration could help predict future temporal ecological variability under reduction in glacial influence^13^.We show that meltwater flow reduction induces changes in the benthic community, characterized by an increase in biomass of benthic algae and macroinvertebrate herbivores. Changes in benthic community composition after flow reduction is 30 times faster than the time required to return to pre-disturbance state after flow recovery. We discuss the ecosystem capacity to recover from meltwater flow reduction and emphasize the importance of understanding how glacier retreat will affect whole alpine freshwater ecosystems in a rapidly changing world.

## Results

### Abiotic and biotic changes under flow alteration

**Figure 1:**
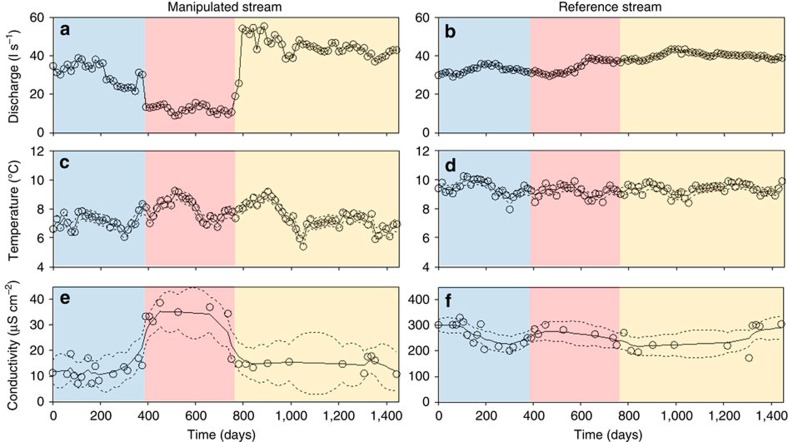
Environmental time series. Time series (open dots), smoothed-state estimates (solid lines) and state 95% confidence level (dotted lines) of discharge (**a**,**b**), water temperature (**c**,**d**), and conductivity (**e**,**f**) for the downstream reach of the manipulated stream and the reference stream. Blue, red and yellow regions indicate base flow, flow alteration and flow recovery periods, respectively.

**Figure 2:**
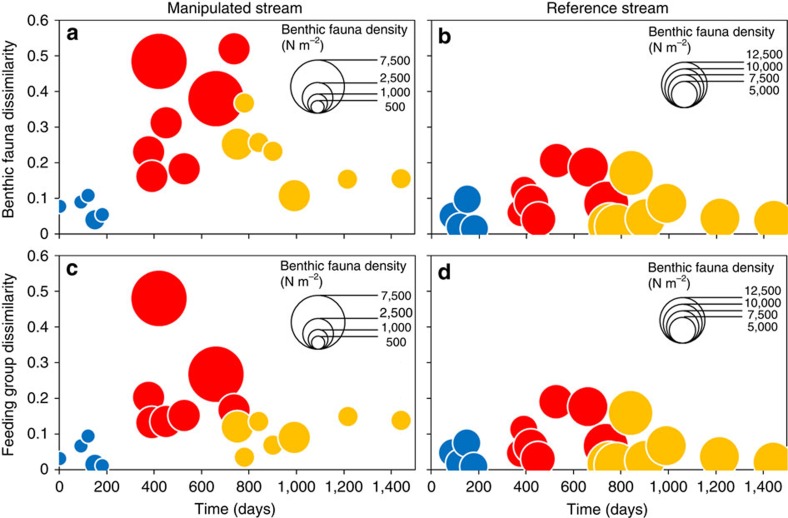
Temporal variability in benthic fauna and feeding group dissimilarity between reaches of the manipulated and reference stream. Benthic fauna and feeding group dissimilarity between downstream and upstream reaches in the manipulated stream (**a**,**c**) and between subsequent sampling dates in the reference stream (**b**,**d**). The three experimental phases are shown in different colours (blue: base flow; red: flow alteration; yellow: flow recovery). Dot size refers to benthic fauna density. Note difference in dot size in the reference and manipulated streams due to much higher overall density in the reference stream.

Our experimental water diversion significantly reduced the mean daily flow by 31% in the manipulated stream and the flow coefficient of variation by 20% (Fig. 1a and Supplementary Table 1). The most marked abiotic changes in the manipulated reach were an increase of 1.4 °C in mean water temperature (Fig. 1c) and a 65% increase in mean conductivity (from 14.4 to 32.2 μS cm^−2^) (Fig. 1e). These abiotic changes were not observed in the reference stream (Fig. 1b,d,f) or in the upstream reach of the manipulated stream (Supplementary Fig. 2). In terms of biota, flow reduction had a strong impact on benthic fauna community composition: dissimilarity between lower and upper stream reach communities rapidly (<15 days) increased following the flow reduction (Fig. 2a). Benthic communities remained relatively stable (that is, dissimilarity below 0.2) in the reference stream, although slight changes in the abundance of a few groups (for example, Hyalellidae, Baetidae and Simuliidae) occurred during the period of low flow in the manipulated stream (for example, increase in Hyalellidae and Simuliidae at day 553, and in Hyalellidae and Baetidae at day 664) and recovery phase (for example, decrease in Hyalellidae and increase in Baetidae at day 864; Fig. 2b). Flow reduction increased benthic fauna density by 6.5 times on average (±1.8) but had no effect on richness. After flow returned to pre-disturbance values, it took the faunal community 14–16 months to return to its original composition, that is, within the confidence interval limits of the natural variability in taxon assemblages (Fig. 2a,b). Changes in benthic faunal assemblages during flow alteration were mainly due to increased abundances of herbivorous/detritivorous Diptera (Orthocladiinae and Ceratopogonidae), Ephemeroptera (Baetidae) and Coleoptera (Elmidae), which were the most significant indicator taxa of post-flow diversion samples (Simper analysis). Likewise, dissimilarity in feeding trait composition between upstream and downstream reaches of the manipulated stream was higher during flow alteration (Fig. 2c), while the feeding trait composition remained constant in the reference stream (Fig. 2d). In the manipulated stream, herbivores had by far the greatest contribution to dissimilarity in feeding trait composition among experimental phases, with higher densities during the low-flow period. Collector–gather and predator densities were also higher during flow alteration but they had an overall small contribution to the observed trophic changes (Table 1).

### Algal and herbivore biomass shifts along flow manipulation

**Figure 3:**
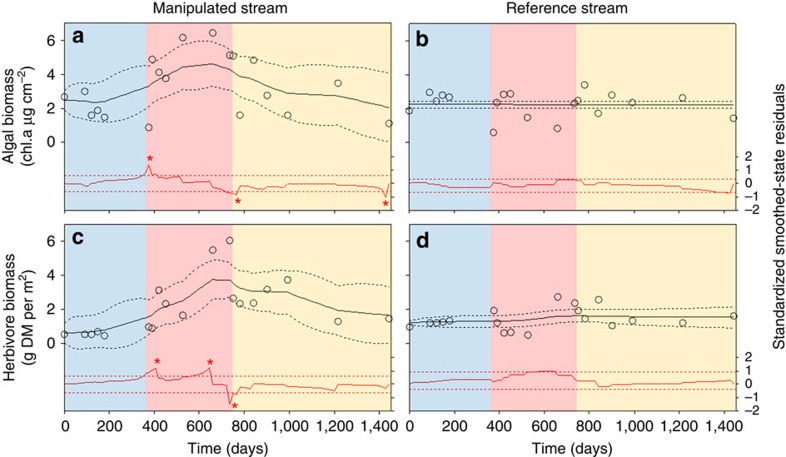
Ecological time series. Time series (open dots), smoothed-state estimates (solid lines) and state 95% confidence level (dotted lines) of algal biomass (chl.a, chlorophyll a; **a**,**b**) and herbivore biomass (DM = dry mass; **c**,**d**) for the downstream reach of the manipulated stream and the reference stream. Red curves represent standardized smoothed-state residuals from state-space models for algae and herbivores time series. Dashed red lines are the 95% confidence intervals for a *t*-distribution. Red stars indicate when standardized smoothed-state residuals are beyond the dashed level lines. Blue, red and yellow regions indicate base flow, flow alteration and flow recovery periods, respectively.

One of the major trophic impacts of the decrease in glacier runoff was the strong increase in the biomass of benthic primary producers (algae) and herbivorous invertebrates (Fig. 3a,c). State-space analyses revealed three significant shifts (large standardized smoothed-state residuals, red stars in Fig. 3a,c) in algal biomass in the manipulated reach: algal biomass increased significantly 2 weeks after flow reduction, decreased 40 days after flow recovery and then remained at intermediate levels up to a final decrease to the initial level, 20 months after flow recovery. Three shifts occurred in herbivore biomass: it increased significantly after 2 weeks of water diversion, reached maximum values at the end of the flow reduction period and decreased to intermediate levels 1 week after flow recovery. Herbivore biomass then slowly decreased to initial levels 14 months after flow recovery. We found no significant shifts in algal and herbivore biomass in the reference stream (Fig. 3b,d). Multivariate autoregressive state-space analysis on algal and herbivore biomass showed significant top–down and bottom–up effects between algae and herbivores in the downstream reach of the manipulated stream (negative effect of herbivores on algae, *B*_h*→*a_=−0.044 and positive effect of algae on herbivores *B*_a*→*h_=0.014, corrected Akaike Information Criterion=83.0), but not in the reference stream (see Supplementary Notes Part 2: 4 for details).

### Ecological shifts across a gradient of glacial influence

**Figure 4:**
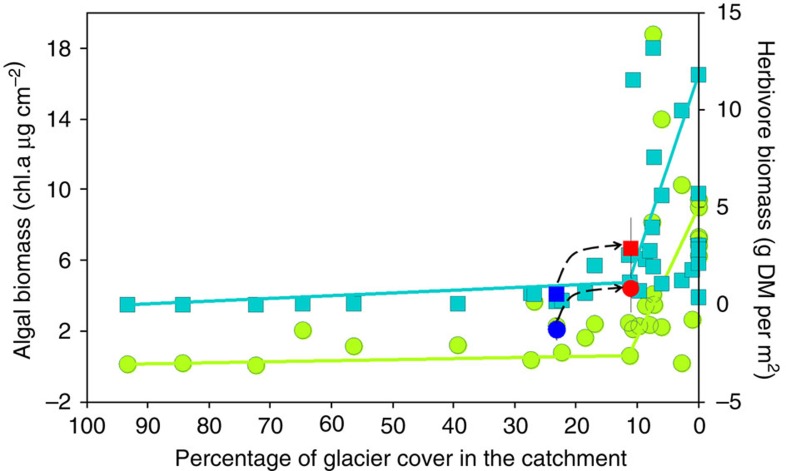
Spatial variability in algae and herbivores along a glacier influence gradient. Relationship between algal (chl.a, chlorophyll a; green dots) and herbivore biomass (blue squares) as a function of the percentage of glacier cover in the catchment for 33 streams in the Antisana reserve (Ecuador). Douglas–Peucker (DP) models are fitted to algal and herbivore biomass. Data (mean 95% confidence interval) of algal (dots) and herbivore (squares) biomass obtained in our flow-reduction experiment are shown in blue (base flow) and red (low flow). The dashed line represents the shift from low to high level of algal and herbivore biomass between base flow and flow alteration stages.

These experimental results were supported by our complementary field survey of 33 stream sites covering a gradient in glacial influence. Our data revealed that, between 93.2 and 11.3% of glacier cover in the catchment, both algal and herbivore biomass remained at relatively constant low levels while below that threshold, both algal and herbivore biomass significantly increased (Fig. 4). When plotting our experimental data on Fig. 4 (blue and red dots and squares), we found that algal and herbivore biomass values before and during experimental flow reduction closely matched the pattern observed with the multi-locality survey data.

## Discussion

Previous studies on benthic fauna response to reduced flow have commonly found significant changes in the community structure and function characterized by change in density (either increases or decreases^14^), reduction in taxon richness^15^ and reorganization of the feeding trait assemblage^16^,^17^. The impacts are however variable among studied systems (for example, natural flow regime, water quality and the features of the disturbance imposed^18^,^19^,^20^). Yet, manipulative experiments in glacier-fed systems are rare, especially for whole ecosystems. Our experimental design allows us to evaluate potential shifts in biota in response to meltwater reduction and assess ecosystem resilience after flow recovery to identify the ecosystem capacity to recover from periodic low flows. In glacier-fed stream, we expect an increase in both density and taxon richness of benthic fauna, as the reduction in glacier runoff should reduce the environmental harshness characterizing glacial meltwater, thereby allowing more species to establish^4^,^10^,^21^. We also expect trophic consequences of these structural changes due to the strong connectance in glacier-fed stream foodwebs^22^,^23^,^24^.

While previous surveys have examined spatial and temporal patterns of stream community response to a range of glacial influence^4^,^6^,^7^,^10^ our relatively long-term experiment offers fresh viewpoints for the prediction of aquatic biota response to glacier retreat. Indeed, our study shows that reduction in glacier runoff would have complex consequences for aquatic biota structure and trophic organization via alterations in resources and trophic interactions. Our combined experimental study and survey supports that a reduction in glacier runoff, and associated glacial influence in mountain streams, beyond a threshold of about 11% of glacier cover in the catchment, would induce an abrupt shift in aquatic biota. As additional field evidence, we observed high algal biomass in natural glacier-fed streams during exceptionally low glacial-shrinking periods (for example, La Niña event, S. Cauvy-Fraunié pers. Obs.), a pattern also reported in temperate glacier-fed rivers^25^. Reductions in both flow and glacial influence imply higher water temperature, conductivity and lower turbidity^26^, thereby increasing the overall productivity of stream systems^14^,^25^.

Our experimental flow manipulation revealed that reduced meltwater discharge significantly influenced the structure of benthic communities. In particular, it enhanced herbivore densities, a response that is likely a result of an increase in benthic algal biomass^14^. Flow reduction had no effect on taxon richness, but rather induced benthic taxon replacement characterized by (1) the gain of new taxa (for example, Ceratopogonidae and Empididae) less adapted to the harsh glacial meltwaters^26^,^27^, (2) the loss of taxa (for example, Blephariceridae and Scirtidae) caused by modifications in environmental conditions (for example, loss of fast flow habitat and higher conductivity)^14^,^27^ and an increase in predation and competition pressures^10^,^14^,^28^. Specifically, lowered collector–filterer densities, such as Simuliidae, were probably attributable to the loss of fast-flow microhabitats as water velocity controls the rate of food delivery for filter feeders^29^. Our study therefore strongly suggests that the relationship between disturbance, species composition and species interactions will be a central issue in understanding climate change effects on glacier-fed aquatic systems, and probably in a wide variety of glacially influenced ecosystems^30^,^31^.

Finally, another key finding of our work is that the recovery of benthic fauna communities affected by flow reduction was rather slow compared with previous studies with similar or stronger percentage of flow reduction^19^,^32^. Resilience can however vary greatly according to studied systems and species (for example, their dispersal capacity), the distance to species pools and the magnitude, duration and timing of flow disturbance^33^,^34^,^35^. Nevertheless, compared with the recurrence interval of low-flow events, the time required for recovery might become insufficient for ecosystem resilience between low flows. Indeed, increasingly variable melting flow regimes^2^,^36^ would induce an increase in the frequency of minimum discharge, in terms of both intensity and duration^37^. If low meltwater discharges were rare, benthic communities would have more time to fully recover between each event. In contrast, more frequent reductions in meltwater discharge should prevent the ecosystem from fully recovering between consecutive flow disturbances, with potentially severe but unknown consequences on the aquatic communities^16^. In addition, after a certain amount of glacier volume lost, glacier runoff would undeniably decrease, thereby inducing shifts in benthic communities^27^.

Our study provides unique insights into how glacially influenced rivers can be expected to respond to glacier retreat under global warming and therefore urges to further increase our understanding on how climate change will affect whole ecosystems. We found that reduction in glacier runoff rapidly induces a reorganization of benthic taxon assemblage, which requires a 30 times longer period to return to initial state. Resilience delay may be prolonged by competition exclusion by dominant herbivore taxa that established during low-flow conditions. Benthic communities constitute the bulk of primary and secondary production of alpine stream ecosystems^22^ and have been widely used to indicate changes in environmental conditions not only in systems receiving meltwater contributions^10^,^38^ but in other aquatic systems^39^ as well. Our study also suggests that alpine river biota could be used as key indicators to identify early signals of ecosystem damaging, such as slowing return rates from perturbation. Such shifts are most relevant to concerns of biodiversity conservation and ecosystem services of aquatic systems in both temperate and tropical mountains where many types of human-induced perturbations affect resilience.

## Methods

### Experimental study streams

We studied two alpine streams, one reference and one manipulated, located at 4,100 m above sea level in the Antisana Reserve, Ecuador (0° 29′ 06″ S, 78° 08′ 31″ W). The two streams were located within the catchment of ‘Los Crespos' glacier, which covered an area of about 1.82 km^2^ in 2010, with an average cumulative mass balance trend of −0.6 meters water equivalent per year (m w.e.yr^−1^) (ref. 3). Both streams shared similar morphological characteristics in terms of slope (0.7–1.2%), width (0.9–1.2 m) and depth (20–50 cm). We monitored abiotic and biotic variables (see below) in two reaches (about 20 m) in the manipulated stream and in one reach in the reference stream. The monitoring started in October 2009 to establish baseline conditions over 1 year. In November 2010 (after 380 days) we built a stone dam on the manipulated stream to divert about one-third of the water, a perturbation that mimics predicted reduction of glacier runoff (2–30% of baseline flows) following glacier shrinkage^2^. Stream flow disturbance effects on biota were assessed over 1 year, and in November 2011 the dam was removed to evaluate stream biota recovery (until October 2013, Supplementary Fig. 1). In the manipulated stream, benthic fauna displacements over the ∼200 m separating the two reaches may render them partly dependent. However, unpublished data on upstream migration traps left for 48 h indicated a low level of upstream migration, and we therefore considered the two reaches statistically independent. Our experimental design based on both manipulated and reference streams and upstream–downstream comparisons maximizes the likelihood that the observed patterns are causally related to flow reduction rather than external drivers^11^. The experiment is conducted over 4 years, the equivalent of ca. 20–40 generations of the studied organisms^40^.

### Abiotic and biotic parameter monitoring

Water pressure loggers (Hobo, Onset Computer Corp., USA) were installed over the study period to record water pressure and temperature every 30 min. One more logger was placed outside the streams at 4,100 m a.s.l. to correct for variation in atmospheric pressure. Pressure data were transformed into discharge values as detailed in a previous study^10^. Conductivity (at 25 °C), pH and dissolved oxygen were measured with portable meters (WTW, Germany) on several visits to the study reaches. At 19 dates spread over the study period (at *t*=0, 87, 132, 160, 181, 388, 397, 419, 448, 533, 664, 759, 766, 798, 864, 923, 1,011, 1,238 and 1,471 days), algal biomass was quantified by collecting 9 small pebbles (2–4 cm) at random and extracting chlorophyll a in 96% ethanol for 1–3 days in the dark until further analysis in the laboratory (see ref. 38 for details). Also, five quantitative Surber samples (0.05 m^2^; mesh size 200 μm) were collected randomly from pebble–cobble substratum. All samples were collected between 09:00 and 14:00 and preserved in the field in 70% ethanol. Invertebrates were counted and identified to morphospecies, genus or (sub) family. Functional feeding groups were assigned to each taxon following^41^,^42^. For the most abundant taxa (representing more than 95% of the relative abundance in the three stream sites), functional feeding groups were refined based on the dominant food items identified via gut content analyses of 10 individuals per taxon^43^,^44^. Macroinvertebrate guts were mounted on to microscope slides by removing the foregut following dissection of macroinvertebrates. Gut contents were placed in a drop of water on a microscope slide and secured with a coverslip. Five randomly selected fields within the slide were viewed at × 200 magnification. In each field on the slide, the approximate percentage by area of the five food types identified (diatoms, filamentous algae, fine detritus, coarse detritus and animal matter) was estimated. A percentage of herbivory was assigned to each dominant taxon according to the relative abundance of diatoms and filamentous algae observed in their gut contents (mean for the 10 individuals)^44^. For each sampling date, herbivore biomass was calculated based on the most dominant herbivore taxa density multiplied by their dry mass and their percentage of herbivory assigned. Organisms' dry mass (DM) was calculated from the measured body length (*L*) of each taxon, using the equation DM=*aL*^*b*^ and using the parameters *a* and *b* provided by ref. 45 for each invertebrate family. As we had no data on the rate of algae and invertebrate reproduction, we could not calculate the productivity of our system. We thus used biomass values of both algae and herbivores as a surrogate of standing stocks.

### Data analyses

Dissimilarity in macroinvertebrate assemblage and feeding trait composition between the upstream and downstream reaches of the manipulated stream was calculated for each sampling date using Bray–Curtis dissimilarity index^46^ to assess how flow reduction affected the overall organization of benthic fauna community structure and trophic composition. An indicator species analysis (SIMPER procedure) was used to determine whether particular taxa were indicative of low-flow or recovery conditions, and which traits accounted for the greatest dissimilarity across the three experimental phases in the manipulated reach. The statistical significance of each indicator species was tested using Monte Carlo randomization with 1,000 runs. For comparison, similar analyses were performed on the reference stream to evaluate the natural variability in benthic fauna community structure and trophic composition.

To estimate trends in abiotic and biotic time series, we used state-space analyses, which describe the observed time series by an underlying state process evolving through time^47^,^48^. For each stream site, univariate autoregressive state-space models with Gaussian errors were fitted independently to discharge, water temperature, conductivity, algal biomass and herbivore biomass time series (15 days time step). Smoothed-state estimates (the expected values of the state process based on the maximum-likelihood values of the model parameters) were then computed using the Kalman smoother, and plotted to examine trends in each time series (Supplementary Notes Part 1: 1–3)^47^,^48^,^49^. To identify potential sudden level changes in algal and herbivore biomass time series, standardized smoothed-state residuals (the difference between the smoothed-state estimate at time *t* and *t*−1 standardized by its s.d.) were computed and compared with 95% confidence interval for a *t*-distribution^47^,^50^ (Supplementary Notes Part 1: 3 and 4). To estimate interaction strengths between algae and herbivores, multivariate autoregressive state-space models were fitted to algal and herbivore biomass time series with discharge, water temperature and conductivity time series as environmental covariates^51^,^52^. In those models, the state processes described changes in algal and herbivore biomass based on interactions between the two trophic levels and environmental covariates, while the observed time series were described by the state processes and observation errors associated with incomplete sampling (Supplementary Notes Part 2). All analyses were performed using the *MARSS* package in R (R Development Core Team, 2013, version 3.0.2)^49^. R codes are available in the Supplementary Notes.

### Complementary field survey

Previously collected data on algal and herbivore biomass in 33 stream sites with different glacial influence^53^ (per cent of glacier cover in the catchment (%GCC) ranged from 0 to 93.2) were used to address the relevance of our experimental results. To compare algal and herbivore biomass in the manipulated stream with the 33 natural stream sites, we calculated the corresponding %GCC of the manipulated stream during flow alteration based on the relationship between glacial meltwater discharge and the %GCC of stream sites located along the study glacierized catchment (4,200–4,800 m). Both algal and herbivore biomass values of the 33 stream sites and of the manipulated stream before and during experimental flow reduction were plotted against %GCC. Breaks in the slope of the relationship between algal and herbivore biomass and glacial influence were identified using the Douglas–Peucker algorithm (Matlab version R2010a; The Mathworks Inc., Natick, MA, USA).

### Data availability

All relevant data are available on request from the authors.

## Additional information

**How to cite this article:** Cauvy-Fraunié, S. *et al*. Ecological responses to experimental glacier-runoff reduction in alpine rivers. *Nat. Commun.* 7:12025 doi: 10.1038/ncomms12025(2016).

## Supplementary Material

Supplementary Information Supplementary Figures 1-2, Supplementary Tables 1-2, Supplementary Notes 1-2 and Supplementary References

## Figures and Tables

**Table 1:** Dissimilarity in feeding trait composition.

**Functional feeding groups**	**Contribution**		**Mean density**		
	**BF→FA**	**FA→FR**	**BF**	**FA**	**FR**
*A. Manipulated stream*					
Herbivores	57.82	28.08	647 (231)	4,325 (2,022)	1,878 (348)
Collector–gatherers	6.26	4.0	43 (25)	447 (322)	169 (83)
Predators	5.71	4.25	60 (43)	451 (357)	244 (246)
Collector–filterers	0.83	0.58	60 (42)	51 (47)	31 (29)
Shredders	0.72	0.49	4 (5)	46 (47)	23 (13)
Overall dissimilarity	71.35	37.4			
					
*B. Reference stream*					
Shredders	15.73	14.07	4,969 (245)	7,943 (2,875)	9,847 (1,803)
Herbivores	2.44	2.23	1,203 (235)	1,513 (451)	1830 (245)
Collector–gatherers	1.12	2.73	403 (75)	231 (117)	815 (618)
Predators	0.92	1.01	244 (98)	321 (152)	110 (74)
Collector–filterers	0.67	0.59	111 (23)	150 (220)	87 (71)
Overall dissimilarity	20.88	20.6			

Relative contribution (%) of the five functional feeding groups to the Bray–Curtis dissimilarity (SIMPER analysis) and overall dissimilarity (%) between base flow and flow alteration stages (BF→FA), and flow alteration and flow recovery stages (FA→FR) in the manipulated (A) and reference (B) reach. Mean density (ind m^−2^) and s.d. of the five functional feeding groups for base flow (BF), flow alteration stages (FA), and flow recovery stages (FR) in the manipulated (A) and reference (B) reach.
