# Supplementary material for: Ecological responses to experimental glacier-runoff reduction in alpine rivers
Source: Nat Commun. 2016 Jun 24;7:12025. doi: 10.1038/ncomms12025 (PMC4931009; doi:10.1038/ncomms12025)
Supplement: Supplementary Information — Supplementary Figures 1-2, Supplementary Tables 1-2, Supplementary Notes 1-2 and Supplementary References [file ncomms12025-s1.pdf]

Supplementary Figure 1.

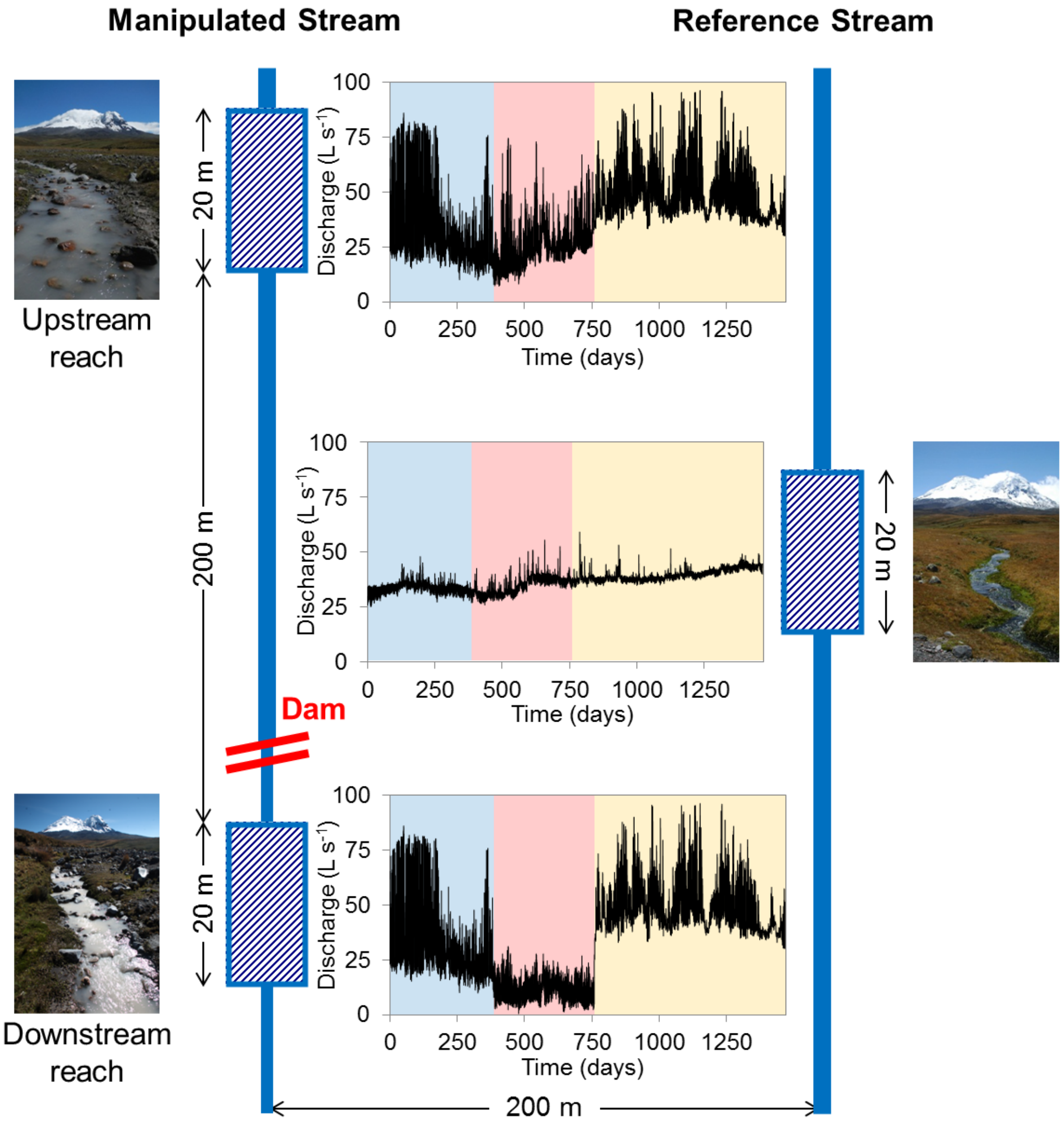

Supplementary Figure 1. Scheme of the field manipulation design. Pictures and temporal patterns of discharge during the flow manipulation experiment were represented for both the manipulated stream (upstream and downstream reaches) and the reference stream. Blue, red and yellow regions indicate base flow, flow alteration and flow recovery periods, respectively. Values on the arrows indicate the length of the stream sites, and the distances among the two streams and the two reaches of the manipulated streams. Photo credits: S.C-F. and O.D.

Supplementary Figure 2.

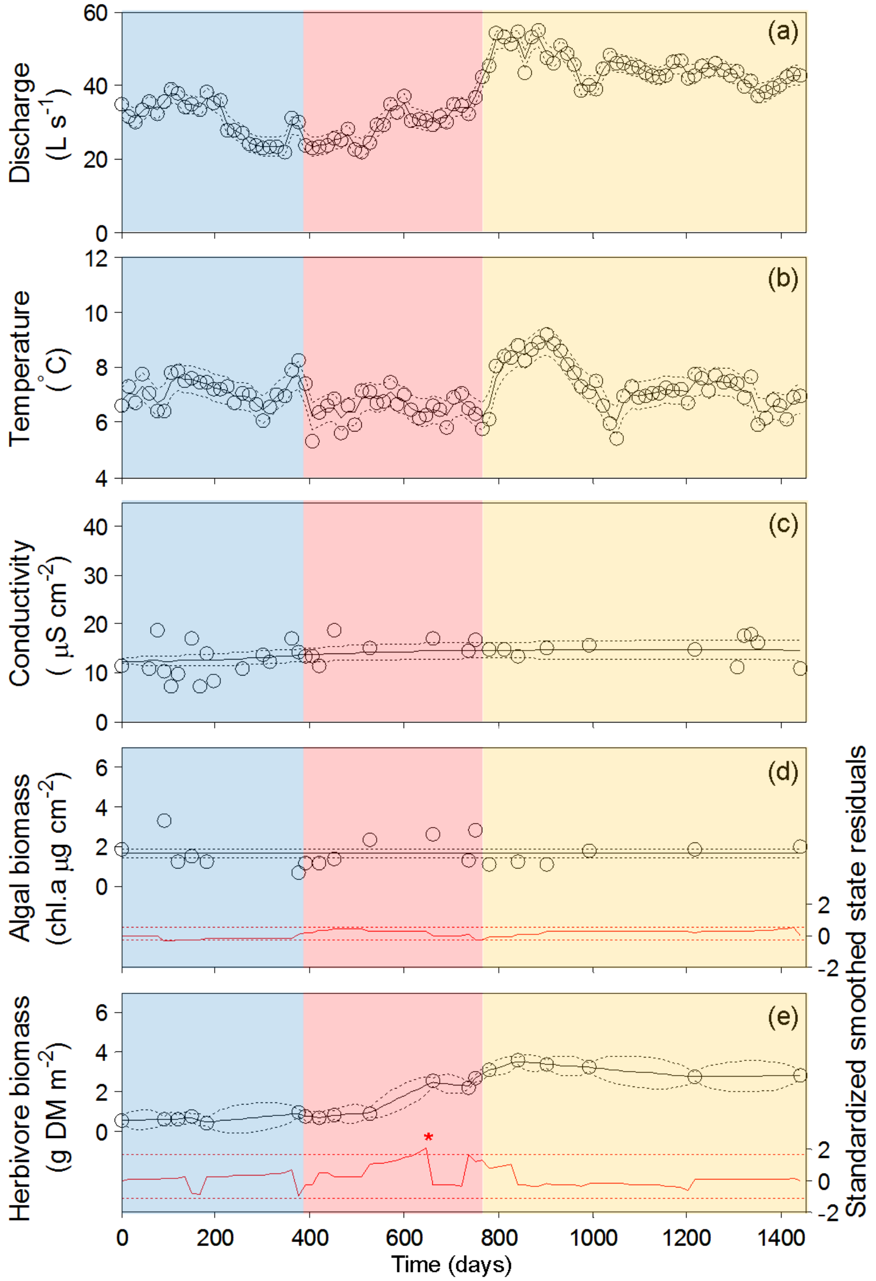

Supplementary Figure 2. Environmental and ecological time series. Times series (open dots), smoothed states estimates (solid lines), and states 95% confidence level (dotted lines) of discharge (a), water temperature (b), conductivity (c), algal biomass (d; chl.a = Chlorophyll *a*), and herbivore biomass (e; DM = dry mass) for the upstream reach of the manipulated stream. Red curves represent standardized smoothed states residuals from state-space models for algal (d) and herbivore biomass (e). The dashed red lines are the 95% CIs for a t-distribution. Red stars indicate when standardized smoothed states residuals are beyond the dashed level lines. Blue, red and yellow regions indicate base flow, flow alteration and flow recovery periods, respectively.

Supplementary Table 1. Abiotic and biotic changes among experimental phases

	Manipulated stream						Reference stream		
	Upstream reach			Downstream reach					
	BF	FA	FR	BF	FA	FR	BF	FA	FR
Discharge	33.7	30.9	31.2	33.7	**21.4***	31.2	31.3	34.5	35.3
	(75.1)	(68.9)	(70.2)	(75.1)	**(55.1)**	(70.2)	(9.8)	(8.9)	(9.1)
Temperature	7.2	6.6	6.7	7.2	**8.0***	6.9	9.4	9.2	9.3
	(0.1-19.7)	(0.1-19.9)	(0.1-18.8)	(0.1-19.7)	(1.5-19.3)	(0.2-18.8)	(3.9-19.8)	(3.5-18.4)	(5.3-18.0)
pH	7.9	8.0	7.7	7.8	7.2	7.4	7.6	7.3	7.2
	(0.4)	(0.2)	(0.3)	(0.3)	(0.5)	(0.5)	(0.2)	(0.1)	(0.5)
Conductivity	13.6	14.4	14.1	13.9	**32.2***	14.7	257.8	269.5	222.1
	(2.7)	(2.2)	(1.3)	(2.8)	(1.6)	(0.7)	(44.3)	(23.3)	(29.7)
Oxygen	106	106	108	107	106	108	131	118	122
	(5.3)	(6.5)	(5.5)	(8.5)	(4.1)	(4.5)	(14.0)	(12.6)	(10.2)
Benthic detritus	14	22	28	24	26	38	44	26	48
	(10-38)	(2-54)	(10-56)	(11-46)	(6-87)	(8-106)	(16-88)	(17-32)	(21-69)
Benthic algae	1.7	1.5	1.7	1.9	**4.5***	2.9	2.2	1.9	2.4
	(1.2-3.3)	(0.7-2.7)	(1.1-2.9)	(1.4-2.9)	(0.8-6.4)	(1.0-5.0)	(1.9-3.3)	(0.6-3.5)	(1.4-3.3)
Benthic fauna richness	30	32	31	31	30	29	29	30	28

Supplementary Table 1. Mean abiotic and biotic variables during the three experimental phases (BF: Base flow, FA: Flow alteration, FR: Flow recovery) in the three experimental reaches in the two study streams (manipulated and reference). Discharges ($L.s^{-1}$) and temperature (°C) are mean daily values provided by automatic loggers. As a measure of parameter variability, we provide the coefficient of variation for discharge (in %), the standard deviation for pH, conductivity ($\mu S.cm^{-2}$), and oxygen (%, n = 8-18), and minimum-maximum values for temperature (°C), benthic detritus (g $DM.m^{-2}$) and algae (µg *Chl*.cm^{-2}). * indicates significant differences in parameter value when compared to values before the experimental flow alteration, in both reference and manipulated streams (significant P values at the 0.05 level from a paired one-tailed t-test).

Supplementary Table 2. Estimation of interaction strengths

Interactions	Manipulated Stream		Reference Stream
	Downstream	Upstream	
Algae → Algae ($B_{a\rightarrow a}$)	1.010 [0.996,1.021]	0.939 [0.898, 0.984]	0.947 [0.899, 1.008]
Herbivore → Herbivore ($B_{h\rightarrow h}$)	0.934 [0.859, 0.967]	0.960 [0.941, 0.987]	0.781 [0.361, 0.933]
Algae → Herbivore ($B_{a\rightarrow h}$)	0.014 [0.001, 0.032]	0.065 [0.020, 0.081]	-0.014 [-0.059, 0.001]
Herbivore → Algae ($B_{h\rightarrow a}$)	- 0.044 [-0.097, -0.008]	0.089 [0.005, 0.171]	0.304 [0.079, 0.679]

Supplementary Table 2. Parameter estimates (maximum likelihood) for interactions between states and bootstrapped 95% confidence intervals for the best model (lowest AICc) for the three study reaches.

Supplementary Notes. State-space model analyses

Supplementary Note 1. Trend estimation

1. *Univariate autoregressive models*

To estimate trends in the environmental conditions (discharge, temperature, and conductivity) and both algal and herbivore biomass time series, univariate autoregressive state-space models with Gaussian errors were fitted independently to each data time series[1-3]. The observed time series Y_t was described by an underlying process, the true state process X_t, evolving through time, as follows.

$$X_t = BX_{t-1} + U + W_t \qquad W_t \sim N(0, Q) \qquad (1)$$

$$Y_t = X_t + V_t \qquad V_t \sim N(0, R) \qquad (2)$$

with X_t a vector of states at time t, Y_t a vector of observations at time t, W_t a vector of process errors (normally distributed with mean 0 and variance Q), V_t a vector of observation errors (normally distributed with mean 0 and variance R). B is the coefficient of autoregression in the state vectors through time. U describes the mean trend.

The vector representation of these models is as follows:

$$[X_i]_t = B_i[X_i]_{t-1} + U_i + [W_i]_t \qquad \text{where } [W_i]_t = N(0, Q_i)$$

$$[Y_i]_t = [X_i]_t + [V_i]_t \qquad \text{where } [V_i]_t = N(0, R_i)$$

with i corresponding consecutively to the discharge (*dis*), water temperature (*temp*), conductivity (*cond*), algal biomass (*a*), and herbivore biomass (*h*) time series.

2. Model selection

For the three environmental time series (discharge, water temperature, and conductivity time series), *B* was set to 1, as we assumed independence between the environmental values at time *t-1* and *t* (15 days after, see[4] for further evidence of this assumption). In an initial model *Q*, *R*, *U* and X_0 were estimated. For all environmental time series, *U* value was estimated close to zero, and thus fixed to 0 in a second model. The best fitting model was identified as having the lowest Akaike Information Criterion adjusted for small sample sizes (AICc)[5-7]. In our case, as the performance of the second model was better than the initial model (lowest AICc), we kept the second model with *U* fixed to 0 to fit our environmental time series ($AICc_{dis}$ = 580, 524, and 281; $AICc_{temp}$ = 153, 188, and 92; $AICc_{cond}$ = 235, 179, 353 for both downstream and upstream reaches of the manipulated stream, and the reference stream, respectively).

For the biota time series, we first evaluated whether algal and herbivore biomass time series showed evidence for density dependence (B ≠ 1) or density independence (B = 1)[7-9]. We, thus, used both density-dependent (*B* estimated) and density-independent (*B* set to 1) models as initial models. In those initial models *Q*, *R*, *U* and X_0 were estimated. For both biota time series, we found similar AICc for the density-dependent and the density-independent models (ΔAICc < 2), suggesting that both models could be considered as equally supported by our data[5]. Longer time series would be required to evaluate whether algae and herbivores showed evidence for density-dependence. However, for both biota time series, AICc was slightly lower for the density-independent model, where *U* was

estimated close to zero (e.g., for the downstream reach of the manipulated stream $AICc_a$ = 84.9, 86.6 and $AICc_h$ = 76.9, 77.6 for the density-independent and dependent models, respectively). In a second model, *U* was thus fixed to 0 and B to 1. We obtained the lowest AICc for this model, and thus fitted algal and herbivore biomass time series with a density-independent model with *U* fixed to 0. ($AICc_a$ = 82, 46, and 49; $AICc_h$ = 74, 28, and 43 for both downstream and upstream reaches of the manipulated stream, and the reference stream, respectively).

3. Smoothed state estimates

Once we selected our model, the smoothed state estimates $\tilde{X}_{t|T}$ at time *t* was computed via the Kalman smoother[10].

$$\tilde{X}_{t|T} = E(X_t|\hat{\theta}, Y_1^T) \qquad (3)$$

The smoothed state estimates $\tilde{X}_{t|T}$ are the expected values of X_t conditioned on the maximum-likelihood values of the model parameters and on the data (*Y* from 1 to *T*); where $\hat{\theta}$ are the maximum-likelihood estimates of the parameters[6,8-9].

4. Detecting sudden level changes

To detect structural breaks in both algal and herbivore biomass time series (when the level of the series shifts up or down), auxiliary residuals were computed from our state-space models[1-2,11-12.] The standardized smoothed state residuals r^*_t are the differences between the smoothed state estimates at time *t* ($\tilde{X}_{t|T}$) and the smoothed state estimates at time *t-1* ($\tilde{X}_{t-1|T}$) standardized by its standard deviation:

$$r_t^* = \frac{\tilde{X}_{t|T} - \tilde{X}_{t-1|T}}{\sqrt{\mathrm{var}(\tilde{X}_{t|T} - \tilde{X}_{t-1|T})}} \tag{4}$$

Auxiliary residuals were considered as a t-test of structural break occurrence, with H0 = there was no structural break in the observed time series[1-2,13]. Sudden changes in level were thus detected when the standardized smoothed state residuals exceed the 95% confidence interval for a t-distribution.

5. R code

State-space analyses were performed using the *MARSS* package in R. In the following script, we provided the code to fit a specific univariate autoregressive model (the AICc best-fitting model in our case with B = 1, U = 0) to a single data time series as well as the calculation of the smoothed state estimates and the 95% confident interval. Note that, in the *MARSS* function, two algorithms, allowing finding the maximum-likelihood estimates of the parameters, are provided: the EM (Expectation-Maximization) and the BFGS (Broyden-Fletcher-Goldfarb-Shanno) algorithm. In our case, we used the BFGS algorithm to polish off the estimates. However, the EM algorithm was used to provide the initial conditions of our models to improve the performance of those algorithms (for details see[8]).

```
# load package MARSS
library (MARSS)
# load the data time series
data=dat
# assign model specification (best model represented)
mod = list (Z=matrix(1), A=matrix(0), B=matrix(1), U=matrix(0), Q=matrix("q"),
R=matrix("r"), x0=matrix("pi"))
# fit the model to the data time series
marss.inits = MARSS (dat, model=mod, method="kem")
marss.fit = MARSS (dat, model=mod, inits=marss.inits$par, method="BFGS")
```

```
# extract the state estimate
Estim_state = marss.fit$states
# calculate the 95% confidence interval of the state estimate
IC1<- Estim_state + 1.96 * marss.fit$states.se
IC2<- Estim_state - 1.96 * marss.fit$states.se
# extract the standardized smoothed state residuals
resids = residuals (mars.fit)$std.residuals[2,]
```

Supplementary Note 2. Estimation of interaction strengths

1. Multivariate autoregressive models

To estimate interaction strengths between algae and herbivores, multivariate autoregressive state-space models with environmental covariates were fitted to both log algal and herbivore biomass time series[14-15]. The observed algal and herbivore biomasses Y_t were thus described by state processes X_t, and observation errors associated with imperfect measurement. The state processes X_t describe changes in biomasses due to ecological interactions, environmental covariates (discharges, temperature, and conductivity), and process errors associated with demographic stochasticity, and unmeasured environmental fluctuations[16-17].

$$X_t = BX_{t-1} + U + Cc_t + W_t \qquad W_t \sim MVN(0, Q) \qquad (5)$$

$$Y_t = ZX_t + V_t \qquad V_t \sim MVN(0, R) \qquad (6)$$

with X_t a matrix of states at time *t*, Y_t a matrix of observations at time *t*, W_t a matrix of process errors (multivariate normal with mean 0 and variance-covariance *Q*), V_t a matrix of observation errors (multivariate normal with mean 0 and variance-covariance *R*). Z is a matrix pairing up observations Y_t to the states X_t. *B* is an

interaction matrix, with self-interaction strengths on the diagonal and inter-specific interaction strengths on the off-diagonals ($B_{i,j}$ is the effect of species *j* on species *i*). c_t is a matrix of environmental covariates at time *t*. *C* is a matrix of coefficients relating the effects of covariates c_t to the states X_t. *U* describes the trend parameter[9,16].

The matrix representation of these models is as follows:

$$\begin{bmatrix} X_a \\ X_h \end{bmatrix}_t = \begin{bmatrix} B_{a\to a} & B_{h\to a} \\ B_{a\to h} & B_{h\to h} \end{bmatrix} \begin{bmatrix} X_a \\ X_h \end{bmatrix}_{t-1} + \begin{bmatrix} U_a \\ U_h \end{bmatrix} + \begin{bmatrix} C_{dis\to a} & C_{temp\to a} & C_{cond\to a} \\ C_{dis\to h} & C_{temp\to h} & C_{cond\to h} \end{bmatrix} \begin{bmatrix} c_{dis} \\ c_{temp} \\ c_{cond} \end{bmatrix}_t + \begin{bmatrix} W_a \\ W_h \end{bmatrix}_t$$

$$\text{where } \begin{bmatrix} W_a \\ W_h \end{bmatrix}_t = MVN\left(0, \begin{bmatrix} Q_a & Q_{cov} \\ Q_{cov} & Q_h \end{bmatrix}\right)$$

$$\begin{bmatrix} Y_a \\ Y_h \end{bmatrix}_t = \begin{bmatrix} Z_a & Z_{ah} \\ Z_{ha} & Z_h \end{bmatrix} \begin{bmatrix} X_a \\ X_h \end{bmatrix}_t + \begin{bmatrix} V_a \\ V_h \end{bmatrix}_t \qquad \text{where } \begin{bmatrix} V_a \\ V_h \end{bmatrix}_t = MVN\left(0, \begin{bmatrix} R_a & R_{cov} \\ R_{cov} & R_h \end{bmatrix}\right)$$

with a, *h*, *dis*, *temp*, *cond*, and *cov* refer to algae, herbivore, discharge, temperature, conductivity, and covariance, respectively. The arrows in subscripts reflect the directionality of the effect.

2. Model selection

Z was set as an identity matrix, as we assumed that each observation time series was associated to one state process. R was modelled as a diagonal and unequal matrix (with zero covariance, R_{cov} = 0), as we assumed observation errors were independent and had different variances (as different sampling methods were used for algae and herbivores). Q was modelled as unconstrained, as we assumed that the variance of process errors was unique for each trophic group and that process errors between the trophic groups were correlated (algae and herbivores

sampled within the same stream)[7,14]. All elements in *B*, *U*, X_0 and *C* were first estimated independently (unconstrained). The most complete model thus included a total of 19 parameters: four in *B* (one pairwise species interactions in both directions and the two autoregressive terms), six in *C* (discharge, temperature, and conductivity independently influencing algal and herbivore biomass), two in *U*, two in X_0, three in *Q* (one process error variance for each trophic level, and one process error covariance between algae and herbivore), and two in *R* (one observation error variance for each trophic level). We computed all possible models from the most complete model (see above) to the simplest model with all elements in *C* and *U* set to zero. The best model was identified as that with the lowest AICc and no convergence issue (conditions in the maximum-likelihood algorithm not encountered) due to the small sample size relative to the number of parameters being estimated[14,18]. Those models were fitted independently for each stream site.

3. Statistical tests

To determine whether or not the models with the greatest support, were appropriate to fit our data, prediction errors were examined for independence, and normality[1-2,14,16]. To evaluate the ability of those final models to explain the overall variation in the biomass time series, we calculated the squared residuals R^2 that describes the proportion of variance explained by the best models[18-19]. Moreover, to assess the uncertainty of the interaction estimated parameters (elements in *B*); bootstrapped confidence intervals of the parameter estimates were computed (for 500 bootstrap samples). Estimated parameters were considered as non-significant

when their corresponding 95% bootstrapped confidence intervals overlapped with zero[14,17-18].

4. Results

For the downstream reach of the manipulated stream, the best model was obtained for $C_{cond\rightarrow a}$ and $C_{cond\rightarrow h}$ estimated (the other *C* elements set to 0), and *U* set to 0 (13 parameters estimated, AICc = 83.0, ΔAICc $_{[worst - best model]}$ = 22.6). For this model, residuals indicated no concerns with the assumption of normality, and no autocorrelation. The overall variation in biomass was better explained for herbivores (R^2 = 0.80) than for algae (R^2 = 0.36). For this model, we found significant interactions among trophic levels: algae had a weak positive effect on herbivores ($B_{a\rightarrow h}$ = 0.014 [0.001; 0.032]), while herbivores negatively affected algae ($B_{h\rightarrow a}$ = - 0.044 [-0.097; -0.008], Supplementary Table 2). For the upstream reach of the manipulated stream, the best model was obtained for $C_{dis\rightarrow a}$ and $C_{cond\rightarrow h}$ estimated, and *U* set to 0 (13 parameters estimated, AICc = 44.3, ΔAICc $_{[worst - best model]}$ = 26.8). However, for this model, the assumption of independence of prediction errors was not satisfied for algae. For the reference stream, the best model was obtained for $C_{dis\rightarrow a}$ estimated, and *U* set to 0 (12 parameters estimated, AICc = 65.8, ΔAICc $_{[worst - best model]}$ = 20.4), but for this model, the assumption of independence and normality of prediction errors was not satisfied.

5. R code

```
# load the species and environmental time series
# log-transform the species time series
dat.bio = log (dat_sp[,1:2])
dat.env = dat_env[,1:3]
```

# assign model specification with environmental covariances (initial model represented)

mod.inter.cov = list (Z="identity", B="unconstrained", A=matrix(0), U=" unconstrained", Q="unconstrained", R="diagonal and unequal", C="unconstrained", c=dat.env, tinitx=1)

# fit the models to the data time series with environmental covariates

marss.inter.cov = MARSS (dat.bio, model= mod.inter.cov)

# extract the estimated parameter of interactions

coef.B.inter.cov = coef (marss.inter.cov, type="matrix")$B

# compute bootstrap 95% confidence for the estimated parameter of interactions

MARSSparamCIs(marss.inter.cov, method="parametric", alpha=.05, nboot=500)

# compute the prediction errors

res.inter.cov = MARSSkfss(marss.inter.cov)$Innov

Supplementary References

1 Durbin, J. & Koopman, S. J. Time series analysis by state space methods. Oxford University Press (2012).

2 Commandeur, J. J. & Koopman, S . J. An introduction to state space time series analysis: Oxford University Press (2007).

3 Holmes, E. E., Ward, E. J. & Wills, K. Marss: Multivariate autoregressive state-space models for analyzing time-series data. *The R Journal* **4**, 11-19 (2012).

4 Cauvy-Fraunié, S., Andino, P., Espinosa, R., Jacobsen, D. & Dangles, O. Temporal scaling of high flow effects on benthic fauna: Insights from equatorial glacier-fed streams. *Limnol. Oceanogr.* **60**, 1836-1847 (2015).

5 Burnham, K. P. & Anderson, D.R. Model selection and multimodel inference: a practical information-theoretic approach: Springer Science & Business Media (2002).

6 Karban. R. & De Valpine, P. Population dynamics of an Arctiid caterpillar–tachinid parasitoid system using state-space models. *J. Anim. Ecol.* **79**, 650-61 (2010).

7 Ward, E. J., Chirakkal, H., González-Suárez, M., Aurioles-Gamboa, D., Holmes, E. E., Gerber, L. Inferring spatial structure from time-series data: using multivariate state-space models to detect metapopulation structure of California sea lions in the Gulf of California, Mexico. *J. App. Ecol*. **47**:47-56 (2010).

8 Dennis, B., Ponciano, J. M., Lele, S. R., Taper, M. L. & Staples, D. F. Estimating density dependence, process noise, and observation error. *Ecol. Monogr.* **76**, 323-341(2006).

9 Holmes, E., Ward, E. & Scheuerell, M. Analysis of multivariate time-series using the MARSS package. *User guide: http://cran.r-project. org/web/packages/MARSS/vignettes/UserGuide.pdf* (2014).

10 Kalman, R.E. A new approach to linear filtering and prediction problems. *J. Basic Eng.* **82**, 35-45 (1960).

11 Harvey, A. C. & Koopman, S. J. Diagnostic checking of unobserved-components time series models. *J. Bus. Econ. Stat.* **10**, 377-389 (1992).

12 Harvey, A., Koopman, S. J. & Penzer, J. Messy time series: a unified approach. *Adv. Econom.* **13**, 103-144 (1998).

13 De Jong, P. & Penzer, J. Diagnosing shocks in time series. *J. Am. Stat. Assoc.* **93**, 796-806 (1998).

14 Ives, A., Dennis, B., Cottingham, K. & Carpenter, S. Estimating community stability and ecological interactions from time-series data. *Ecol. Monogr.* **73**, 301-330 (2003).

15 Hampton, S. E., Holmes, E. E., Scheef, L. P., Scheuerell, M. D., Katz, S. L., et al. Quantifying effects of abiotic and biotic drivers on community dynamics with multivariate autoregressive (MAR) models. *Ecology*. **94**, 2663-2669 (2013).

16 Hinrichsen, R. A. & Holmes, E. E. Using multivariate state-space models to study spatial structure and dynamics. in *Spatial ecology* (eds Cantrell, S., Cosner, C., & Ruan, S.) 145-166 (CRC Press, 2009).

17 Ruhí, A., Holmes, E. E., Rinne, J. N. & Sabo, J. L. Anomalous droughts, not invasion, decrease persistence of native fishes in a desert river. *Global Change Biol*. **21**, 1482-1496 (2015).

18 Hampton, S. E., Scheuerell, M. D. & Schindler, D.E. Coalescence in the lake washington story: Interaction strengths in a planktonic food web. *Limnol. Oceanogr.* **51**, 2042-2051 (2006).

19 Ives, A., Carpenter, S. & Dennis, B. Community interaction webs and the response of a zooplankton community to experimental manipulations of planktivory. *Ecology*. **80**, 1405-1421 (1999).
